# Couple-Focused Smartphone Intervention to Reduce Problem Drinking: Pilot Randomized Control Trial

**DOI:** 10.2196/58622

**Published:** 2024-11-01

**Authors:** David H Gustafson Sr, David H Gustafson Jr, Marie-Louise Mares, Darcie C Johnston, Olivia J Vjorn, John J Curtin, Elizabeth E Epstein, Genie L Bailey

**Affiliations:** 1 Center for Health Enhancement Systems Studies University of Wisconsin–Madison Madison, WI United States; 2 Department of Industrial and Systems Engineering University of Wisconsin–Madison Madison, WI United States; 3 Department of Communication Arts University of Wisconsin–Madison Madison, WI United States; 4 Department of Psychology University of Wisconsin–Madison Madison, WI United States; 5 Department of Psychiatry University of Massachusetts Chan Medical School Worcester, MA United States; 6 Department of Psychiatry & Human Behavior Warren Alpert Medical School Brown University Providence, RI United States; 7 Stanley Street Treatment and Resources Fall River, MA United States

**Keywords:** alcohol use disorder, AUD, mHealth, mobile health, mobile phone, smartphone, couple therapy, Comprehensive Health Enhancement Support System, A-CHESS, Alcohol Behavioral Couple Therapy, ABCT

## Abstract

**Background:**

Alcohol use disorder is among the most pervasive substance use disorders in the United States, with a lifetime prevalence of 30%. Recommended treatment options include evidence-based behavioral interventions; smartphone-based interventions confer a number of benefits such as portability, continuous access, and stigma avoidance; and research suggests that interventions involving couples may outperform those for patients only. In this context, a behavioral intervention delivered to couples through smartphones may serve as an effective adjunct to alcohol use disorder treatment.

**Objective:**

This pilot study aimed to (1) evaluate the feasibility of comparing a patient-only (Addiction version of the Comprehensive Health Enhancement Support System; A-CHESS) versus a couple-focused (Partner version of the Comprehensive Health Enhancement Support System; Partner-CHESS) eHealth app for alcohol misuse delivered by smartphone, (2) assess perceptions about and use of the 2 apps, and (3) examine initial indications of differences in primary clinical outcomes between patient groups using the 2 apps. Broadly, these aims serve to assess the feasibility of the study protocol for a larger randomized controlled trial.

**Methods:**

A total of 33 romantic couples were randomized to 6 months of A-CHESS app use (active treatment control) or Partner-CHESS app use (experimental). Couples comprised a patient with current alcohol use disorder (25/33, 76% male) and a romantic partner (26/33, 79% female). Patients and partners in both arms completed outcome measure surveys at 0, 2, 4, and 6 months. Primary outcomes were patients’ percentage of days with heavy drinking and percentage of days with any drinking, measured by timeline follow back. Secondary outcomes included app use and perceptions, and multiple psychosocial variables.

**Results:**

At 6 months, 78% (14/18) of Partner-CHESS patients and 73% (11/15) of A-CHESS patients were still using the intervention. The apps were rated helpful on a 5-point scale (1=not at all helpful, 5=extremely helpful) by 89% (29/33) of both Partner-CHESS patients (mean 3.7, SD 1) and partners (mean 3.6, SD 0.9) and by 87% (13/15) of A-CHESS patients (mean 3.1, SD 0.9). At 6 months, Partner-CHESS patients had a nonsignificantly lower percentage of days with heavy drinking compared with A-CHESS patients (β=–17.4, 95% CI –36.1 to 1.4; *P*=.07; Hedges *g*=–0.53), while the percentage of drinking days was relatively equal between patient groups (β=–2.1, 95% CI –24.8 to 20.7; *P*=.85; Hedges *g*=–0.12).

**Conclusions:**

Initial results support the feasibility of evaluating patient-only and couple-focused, smartphone-based interventions for alcohol misuse. Results suggest that both interventions are perceived as helpful and indicate maintained engagement of most participants for 6 months. A future, fully powered trial is warranted to evaluate the relative effectiveness of both interventions.

**Trial Registration:**

ClinicalTrials.gov NCT04059549; https://clinicaltrials.gov/ct2/show/NCT04059549

## Introduction

### Background

In 2021, more than 28.6 million US adults aged 18 and older, nearly 1 in 9, met the criteria for alcohol use disorder (AUD) [1-3], and in 2020, the first year of the pandemic, alcohol-related deaths increased 25.5% over the previous year [4]. While the pandemic greatly exacerbated the problem, AUD was already epidemic in its proportions. In 2019, some 14.1 million, or 5.5% of the adult population, met criteria for the disorder [5]. In 2018, the American Psychiatric Association’s practice guideline for AUD treatment reported a 12-month AUD prevalence among US adults of 14% and a lifetime prevalence of 30% [6]. According to the National Institute on Alcohol Abuse and Alcoholism (NIAAA), the rate of alcohol-related emergency department visits between 2006 and 2014 increased by 47% [4].

Despite these numbers and the devastating personal, social, and economic impact they represent, AUD is severely undertreated. The 2019 National Survey on Drug Use and Health reported that 7.3% of US adults with AUD received treatment that year [7], a figure that dropped to 4.6% in 2021 [3]. While the pandemic intensified both drinking and lack of treatment [8,9], AUD has long been a substance use crisis in the United States, second only to tobacco use in prevalence.

Current NIAAA guidelines emphasize that treatment options are not confined to traditional peer support or clinic-based outpatient or residential care [10]. Rather, treatment should consist of a personalized combination of evidence-based behavioral treatments, medications, and mutual support groups. Within this context, the use of smartphone interventions as a treatment adjunct may offer certain benefits, including greater accessibility and affordability relative to clinic-based care. In addition, they offer continuous rather than intermittent access to support due to their portability, and because they do not require in-person interaction, they may feel less stigmatizing or stressful than 12-step meetings or clinic sessions [11,12]. Several systematic reviews indicate that smartphone interventions can improve outcomes for individuals with substance use disorders [13,14], including AUDs [15,16].

The novel feature of the smartphone intervention tested in this study is that it provides resources and support not only for individuals with AUD but also for their romantic partners. The premise is that alcohol problems, relationship problems, and maladaptive communication are intertwined and that working to address these issues simultaneously can improve drinking-related outcomes and psychosocial functioning and relationship satisfaction of both partners [17]. A 2020 meta-analysis of 16 randomized controlled trials (RCTs) comparing interventions that were focused on the patient alone (eg, cognitive behavioral therapy and 12-step programs) versus those that also included a partner (eg, alcohol behavioral couple therapy, family therapy, and community reinforcement approach) found that partner-inclusive interventions were associated with larger reductions in substance use and substance-related problems (Cohen *d*=.24) [18]. However, a 2022 review focused on the implementation of behavioral couple therapy for romantic dyads noted the challenges of implementing couple-focused interventions that involve in-person, multisession therapeutic counseling [19]. The bar for entrance into these interventions tends to be high in terms of geographic access, money, time, and commitment from both partners.

Thus, the key question of this project is whether the ease and accessibility of a smartphone intervention for individuals with AUD could be productively married with the benefits of couple-focused therapy. Doing so may not only reduce logistical barriers to the intervention material but also offer opportunities to leverage the phone’s communicative functions. Research with young romantic dyads found that those who used smartphones more tended to communicate with each other more often and in more affectionate ways [20]. Moreover, texting offers accessibility and editability in reaching out to a partner for support at moments of craving and in providing this support. As such, a smartphone dyadic intervention could not only provide each partner with content to build motivation and tools for coping but might also facilitate more timely, effective communication between them.

### Overview of the Intervention

The intervention tested in this study integrates the Addiction version of the Comprehensive Health Enhancement Support System (A-CHESS), an existing smartphone app that helps individuals work toward reduced alcohol use, with key components of Alcohol Behavioral Couple Therapy (ABCT), an outpatient addiction treatment protocol for couples, to create a new app, Partner version of the Comprehensive Health Enhancement Support System (Partner-CHESS), for couples to use together.

### A-CHESS Smartphone Intervention

A-CHESS is a smartphone intervention for reducing alcohol use [21]. Designed to enhance coping competence, intrinsic motivation, and social relatedness, it is consistent with the principles of self-determination theory [22], with services that include a moderated peer support discussion board, tools to ease distress (eg, guided meditation and games), curated information about the patient’s condition and treatment options, and tools for self-tracking such as a weekly check-in which users are prompted in the app to complete. A-CHESS has additional features specific to addiction, such as personalized reminders of recovery motivations, geolocation functions to warn individuals when they are near predesignated high-risk areas (eg, a bar where they used to drink), and tools for reaching support in moments of crisis.

A-CHESS has an established evidence base. In an RCT with individuals exiting residential treatment (N=349), A-CHESS, compared with usual care, reduced risky drinking days by 47% and improved abstinence by 23% [21]. A field test in Appalachia (n=198) found that women with substance use disorders, including alcohol, who were given A-CHESS averaged more than twice as many 15-minute treatment service units and remained in treatment more than 50% longer relative to a nonrandomized comparison group [23]. In an RCT that examined whether drinking outcomes were improved by supplementing an intensive outpatient AUD program with continuing care delivered remotely to a sample of predominantly Black men (n=262), the percentage of heavy drinking days was 34% lower for patients using A-CHESS versus those receiving usual care [24].

### ABCT Protocol

ABCT is a comprehensive, stand-alone, outpatient, couples-based protocol with an extensive evidence base dating from 1986 to 2023 [17,25,26]. Weekly sessions focus on teaching patients with AUD and their partners adaptive coping skills to improve couple and family interactions, address high-risk situations for drinking, reinforce abstinence, prevent relapse, and improve each partner’s well-being [27,28]. ABCT has demonstrated efficacy with various samples of alcohol-dependent, drug-dependent, and dual-diagnosis adult males and females to increase abstinence rates, decrease alcohol- and drug-related problems, and improve couple and family relationship functioning [29]. It has been extended with preliminary positive findings to a brief add-on model for an addiction-intensive outpatient program [30]. Targeted outcomes of ABCT include treatment engagement, reduced or eliminated substance use during and after treatment, reduction in psychiatric distress and symptoms of the patient and partner, improved quality of life for patient and partner, improved couple and family functioning and communication, and better coping skills.

As mechanisms of behavior change, hypothesized active ingredients of ABCT include cognitive behavioral, alcohol-focused, patient interventions; cognitive behavioral, alcohol-focused, partner-related interventions; relationship enhancement; and common therapeutic factors [17,31]. These treatment components are designed to impact four hypothesized mediators of the identified patient’s drinking outcomes: (1) patient and partner motivation, (2) patient and partner coping skills, (3) the support of the partner for the patient’s change efforts, and (4) positive relationship behaviors, with some evidence to suggest that positive dyadic behaviors predict better drinking outcomes for the patient [32]. In addition, studies of in-session verbal behaviors of couples in ABCT that predict positive drinking outcomes suggest that this line of research is fruitful in understanding the mechanisms of drinking and relationship improvement [31,33-35].

### Partner-CHESS: Building on A-CHESS and ABCT

In addition to all features of A-CHESS, Partner-CHESS includes a daily check-in for self-tracking of drinks, cravings, and mood in the patient version, or worry and mood in the partner version, as well as a series of 6 e-learning modules based on core elements of ABCT. The 5- to 10-minute modules teach the ABCT skills to couples, which include good communication skills, identifying and planning for triggers, cravings discussion, handling slips and relapses, noticing something nice, and partners supporting abstinence. In the original, in-person ABCT intervention, the therapist leads the couple through the curriculum at successive meetings; the in-app Partner-CHESS modules are self-guided and may be completed by the couple working together or separately.

The cravings module, for example, teaches couples to discuss effective ways for the patient to ask for help with a craving (eg, encouraging the patient to come home directly and watch a movie together instead of stopping at a bar on the way home) and helpful ways for the partner to respond. They then enter agreed-upon statements into the app; these statements can be texted to each other in moments of craving, using the app’s texting feature. Similarly, after agreeing in the relapses module on a joint plan for responding constructively to a slip or relapse, couples then enter the plan into the app to be shared in the moment of need through PartnerLink.

### Study Objectives

We conducted a pilot RCT comparing Partner-CHESS and A-CHESS with the goals of (1) refining and assessing the feasibility of the study protocol (including our ability to recruit and retain dyads, the reliability and completeness of measures, and the engagement of participants with the interventions), (2) obtaining participants’ ratings of the perceived helpfulness of each intervention, and (3) conducting analyses that explore preliminary indications of differences in outcomes between the Partner-CHESS and A-CHESS arms. The A-CHESS arm was conceptualized as an active treatment control while testing Partner-CHESS allowed us to explore whether integrating elements of ABCT enhanced the effectiveness of the A-CHESS app. The 2 primary outcomes for testing preliminary differences in outcomes between the arms were (1) patients’ percentage of days with heavy drinking and (2) patients’ percentage of days with any drinking. We chose a heavy drinking outcome because substantial evidence suggests markedly more negative consequences of persistent heavy drinking than of low-risk drinking to health, health care costs, and morbidity [36-38]. At the same time, the percentage of drinking days has long been a standard outcome in rigorous alcohol use studies, allowing the measure of reduction in drinking frequency (including, but not necessarily, complete abstinence) [39]. We also performed descriptive analyses to explore several secondary outcomes including aspects of relatedness, motivation, and coping competence, constructs identified by self-determination theory as contributing to the adaptive functioning of individuals [22].

## Methods

### Trial Design

For this nonblinded, pilot RCT, couples were allocated 1:1 to 6 months of either A-CHESS (an active treatment control) or Partner-CHESS, with research assessments at baseline and 2 , 4, and 6 months for patients and partners in both arms. Participants given a study app had used their app for 6 months.

### Participants

To be eligible, the drinker, identified as “patient” in the study, either met criteria for a *Diagnostic and Statistical Manual of Mental Disorders, Fifth Edition* (*DSM-5*) diagnosis in the past year of at least mild AUD (score of 2 or more on an 11-item self-report checklist [40,41]) or currently met NIAAA guidelines for heavy drinking (more than 3 standard drinks in a typical day or 7 in a typical week for women; more than 4 standard drinks in a day or 14 in a week for men [42]). In addition, the patient was married or in a committed romantic relationship for at least 6 months with a partner willing to participate; cohabitation was not required. Patients and partners both had to be aged 18 years or older. Exclusion criteria for patients and partners were having a self-reported mental or physical condition that would limit the use of a smartphone, having a fear of being in the study (eg, triggering intimate partner violence), having a history of schizophrenia, and reporting illicit opioid use on at least 4 of 7 days in a typical week over the last month. Partners were not excluded on the basis of their alcohol use.

### Recruitment and Randomization

Couples were recruited through multiple avenues, such as advertisements on Craigslist and Facebook in Wisconsin and the northeastern United States; a mass email sent to faculty and staff at the University of Wisconsin-Madison; and flyers distributed at a treatment clinic in Madison and a clinic in Fall River, Massachusetts. We had originally planned to recruit only through clinics, but the COVID-19 pandemic, which coincided with the beginning of recruitment, caused us to pivot to an online community strategy of social media ads and email.

Either the self-identified patient or the partner contacted the study staff, and both members of the couple responded to eligibility questions in a telephone interview. If eligible, study information sheets, consent forms, and copies of the patient and partner baseline surveys were mailed to the couple. Study staff obtained verbal consent and completed baseline surveys through telephone with the patient and partner separately, with all participants reading their hard copy during the assessment to facilitate comprehension.

Upon completing the baseline survey, study staff used a computer-generated allocation sequence to assign couples 1:1 to Partner-CHESS or A-CHESS. Couples were dynamically randomized during the rolling recruitment process, with assignment stratified by the patient’s gender and the couple’s site, using 6 strata established at the start of recruitment: female from Massachusetts, female from Wisconsin, male from Massachusetts, male from Wisconsin, nonbinary from Massachusetts, and nonbinary from Wisconsin. We sought an even gender distribution in the study arms because there are known gender differences in patterns of AUD [43]. Groups were continuously monitored to maintain balance to the extent possible with a small sample and 6 strata. A total of 33 couples received treatment, 15 in the A-CHESS arm and 18 in Partner-CHESS. This sample size falls within the guidelines for pilot study examination of feasibility and adequacy of instrumentation [44].

Because A-CHESS was given only to the patient in a couple while Partner-CHESS was given to both partners, it was not possible to blind couples to their condition. Study staff were blinded at baseline, before randomization, but not at subsequent surveys, which, like the baseline surveys, were conducted by telephone.

Each participant received US $20 for each of the 4 completed surveys. In addition, participants received either US $50 per month toward phone service or, if needed, a smartphone plus service for the duration of the study.

Recruitment ran from October 2020 to March 2021. Final surveys were completed in September 2021.

### Study Arms

Patients in the A-CHESS arm received the A-CHESS app and, if needed, a smartphone. They then participated in a training call, during which study staff confirmed that the app was installed and described the various services and how to use them. The partner did not receive an intervention or smartphone but was expected to participate with the patient as usual, in terms of communication, support, and involvement.

In the Partner-CHESS arm, patients and partners each received a version of the app designed for their role in the couple. They each received a smartphone, if needed, and participated in the training call, together when possible.

Partners and patients in the A-CHESS arm were not discouraged from talking about the intervention content with each other. Rather, A-CHESS was analogous to any other form of care or self-help provided to patients that does not specifically include content for the partner or try to engage the partner. For example, patients who attend 12-step meetings may well discuss them with their partner, and partners may well encourage the patient to attend meetings. In contrast, the Partner-CHESS arm was more analogous to couple therapy for AUD, in that both patient and partner received content and the intent was to facilitate and improve the dyad’s communication about that content.

### Measures

#### Primary Outcomes

At each time point, a timeline follow back interview [45] was completed for the past 60 days. This was used to calculate the 2 outcomes, which are the patient’s percentage of heavy drinking days during the period (“percent heavy drinking days”) and the percentage of days with any drinking during the period (“percent drinking days”). Heavy drinking was defined as more than 4 standard drinks in 1 day for men and more than 3 standard drinks in 1 day for women [40].

#### Secondary Outcomes

Psychosocial outcomes related to relatedness, motivation, and coping were assessed at each time point. Measures were selected for good psychometric properties with similar populations and because they represent pertinent intervention targets and hypothesized mechanisms of change. These secondary outcomes include measures of relationship satisfaction (Dyadic Adjustment Scale-Brief [46-48]), perceptions of family environment (Cohesion and Conflict subscales from the Family Environment Scale [49]), psychological distress (Outcome Questionnaire-45 [50,51]), sobriety motivation (Commitment to Sobriety Scale [52]), partner’s peer support (McTavish Bonding Scale [53]), and partner’s coping strategies (Partner Interaction Questionnaire, adapted for drinking rather than smoking [54-56]). Additional detail regarding these measures is provided in the Multimedia Appendix 1 [46-56].

#### App Use

Smartphones continuously collected all participants’ use of the A-CHESS and Partner-CHESS apps, including clicks into various features and pages as well as text entered into discussion threads or message functions. In addition, at the study conclusion, A-CHESS patients and Partner-CHESS patients and partners rated the overall helpfulness of the app they had used, as well as the helpfulness of individual features, on a Likert scale (1=not at all helpful, 5=extremely helpful).

### Statistical Methods

We performed all analyses using R Statistical Software (R Core Team) within RStudio [57]. Linear mixed-effects models were estimated using the *lme4* package [58], with *P* values provided by the *car* package [59].

We report descriptive statistics (mean and SD for quantitative measures; counts [n] and percentages [%] for categorical measures) for baseline characteristics (eg, age and the number of AUD symptoms) by study arm. For feasibility objectives, n (%) values are used to assess recruitment, reliability statistics are reported for survey measures, and mean and SD are used to assess the app use of participants and perceptions.

We used separate linear mixed-effects models to assess the effect of the study arm for each of the 2 drinking-related primary outcomes, following established guidelines for the use of these models in the social sciences [60]. For each model, we regressed the outcome on the study arm, postintervention time point, and the study arm intervention time point interaction. Baseline scores on the outcome were mean-centered and included as a covariate to control for baseline differences and increase power [61]. No other covariates were included. The study arm was coded using unit-scaled, centered coefficients (ie, 0.5 vs –0.5 for Partner-CHESS vs A-CHESS). Time point was treated as continuous and centered at 4 months (ie, –2, 0, and 2 for 2, 4, and 6 months after intervention, respectively) such that the main effects of the study arm reflected differences in the intervention at the 4-month time point. We included a by-participant random intercept and by-participant random slope for time point in each of the 2 models. Both models were estimated with restricted maximum likelihood.

Our primary test for differences between the study arms focused on the main effect of the study arm in each of these 2 models. Second, we also examined the parameter estimate for the study arm time point interaction to determine if the magnitude of the study arm effect changed over time. We used Type III Wald *F* tests with Kenward-Roger degrees of freedom to test all parameter estimates. Effects are described as significant if *P*<.05. The 95% CI is provided for all parameter estimates, and Hedges *g* is used to describe the simple study arm effect sizes at each time point because it includes a correction for bias due to small sample sizes [62]. Hedges *g* values of 0.20, 0.50, and 0.80 are considered small, medium, and large effect sizes, respectively [63].

We recognize that small sample sizes increase the probability of type II errors for null results and decrease the positive predictive value for significant results [64]. To address these concerns, we have limited our inferential analyses to only these 2 primary outcomes and focused our inferential tests on only 4 parameter estimates (ie, study arm and study arm time point for the 2 outcomes) across these 2 models. We believe these inferential analyses are useful in this feasibility pilot because they demonstrate the analysis strategy that will be used in a planned larger RCT, and they provide a CI for which a preliminary effect size can be represented. Nonetheless, these statistical tests were considered subordinate to the primary objective of evaluating the feasibility of conducting an RCT of both interventions and conclusions based on these results are made with caution. We report descriptive statistics for secondary outcomes in Multimedia Appendix 1 [46-56].

### Ethical Considerations

The study was approved by the University of Wisconsin-Madison Health Sciences Institutional Review Board (IRB; 2018-0696). As the coordinating center for this multisite study, the University of Wisconsin-Madison Center for Health Enhancement Systems Studies was responsible for obtaining IRB approval and served as the IRB of record for all sites. Weekly conference calls facilitated by the project director provided ongoing monitoring of study progress and ethical oversight. All participants provided consent as described in the *Recruitment and Randomization* section. All data is de-identified.

## Results

### Participants

**Figure 1:**
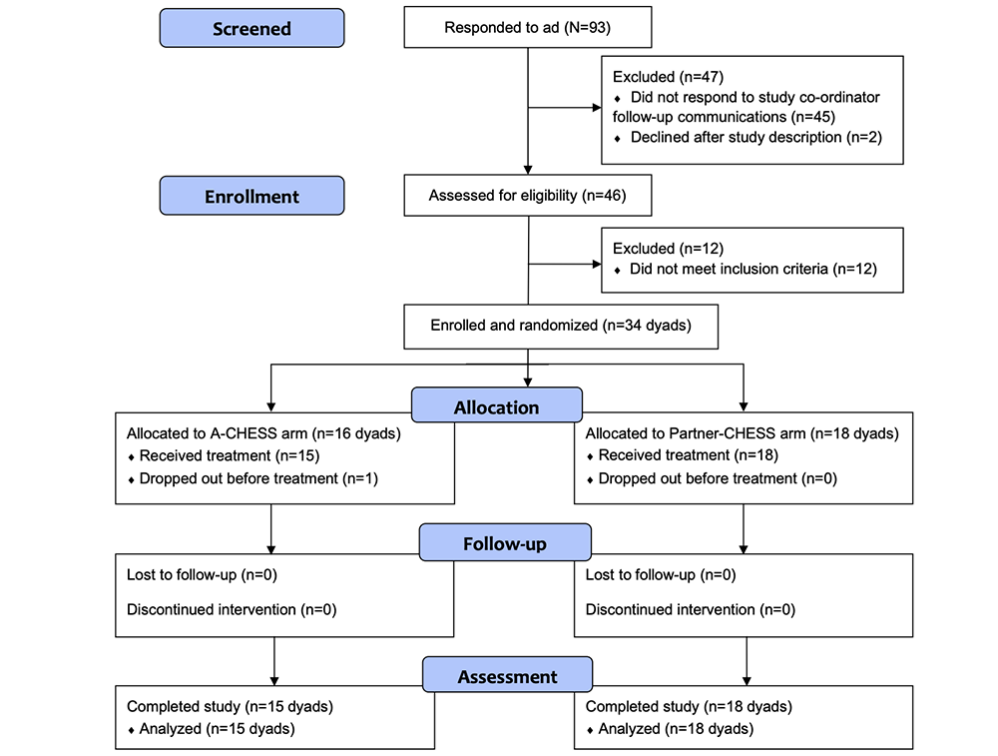
CONSORT (Consolidated Standards of Reporting Trials) flowchart of participants. A-CHESS: Addiction version of the Comprehensive Health Enhancement Support System; Partner-CHESS: Partner version of the Comprehensive Health Enhancement Support System.

A total of 46 couples were assessed for eligibility; 34 met the criteria and were randomized, and 1 assigned to A-CHESS declined to participate before receiving the intervention. Of the 33 couples who participated, 18 (55%) were recruited by email, 13 (39%) by online advertisements, 1 (3%) by treatment clinic, and 1 (3%) by word of mouth. Across both study arms, 80% (53/66) of participants were White, and most patients 76% (25/33) identified as male. All patients met *DSM-5* criteria for at least mild AUD (score of 2 or more on an 11-item self-report checklist [41]); mean scores were 8.27 (SD 2.89) and 9.17 (SD 1.86) for A-CHESS and Partner-CHESS patients, respectively, and 91% (30/33) had a score of 6 or more, signifying severe AUD. Table 1 presents the characteristics of participants at baseline. Figure 1 shows the CONSORT (Consolidated Standards of Reporting Trials) flowchart of participants throughout the study.

**Table 1:** Baseline characteristics of patients and partners by study arm. A total of 33 patient-partner dyads (15 A-CHESS^a^ patients, 15 A-CHESS partners, 18 P-CHESS^b^ patients, 18 Partner-CHESS partners).

Characteristics			Patients						Partners				
			A-CHESS (n=15)		P-CHESS (n=18)		All (n=33)		A-CHESS (n=15)		P-CHESS (n=18)		All (n=33)
Gender, n (%)													
	Female	3 (20)		4 (22)		7 (21)		13 (87)		13 (72)		26 (79)	
	Male	12 (80)		13 (72)		25 (76)		2 (13)		5 (28)		7 (21)	
	Prefer not to say	0 (0)		1 (6)		1 (3)		0 (0)		0 (0)		0 (0)	
Site, n (%)													
	Wisconsin	6 (40)		6 (33)		12 (36)		6 (40)		6 (33)		12 (36)	
	Massachusetts	9 (60)		12 (66)		21 (64)		9 (60)		12 (66)		21 (64)	
Age (years), mean (SD)			43.3 (12.4)		37.8 (10.5)		40.3 (11.4)		40.9 (12.4)		38.5 (10.3)		39.6 (11.2)
Race or ethnicity, n (%)													
	American Indian or Alaskan Native	0 (0)		0 (0)		0 (0)		0 (0)		0 (0)		0 (0)	
	Asian	0 (0)		0 (0)		0 (0)		1 (7)		0 (0)		1 (3)	
	Black or African American	1 (7)		2 (11)		3 (9)		0 (0)		3 (17)		3 (9)	
	Hispanic or Latine	0 (0)		0 (0)		0 (0)		0 (0)		0 (0)		0 (0)	
	Native Hawaiian or Pacific Islander	0 (0)		0 (0)		0 (0)		0 (0)		0 (0)		0 (0)	
	White	12 (80)		14 (78)		26 (79)		12 (80)		15 (83)		27 (82)	
	Multiple races or ethnicities^c^	2 (13)		2 (11)		4 (12)		2 (13)		0 (0)		2 (6)	
Education, n (%)													
	Less than high school	0 (0)		1 (6)		1 (3)		0 (0)		1 (6)		1 (3)	
	High school diploma or equivalent	2 (13)		3 (17)		5 (15)		2 (13)		2 (11)		4 (12)	
	2-year degree or above	13 (87)		14 (78)		27 (82)		13 (87)		15 (83)		28 (85)	
Employed			12 (80)		13 (72)		25 (76)		12 (80)		12 (67)		24 (73)
Living with partner			12 (80)		15 (83)		27 (82)		12 (80)		16 (89)		28 (85)
Experienced partner violence in the past 4 months			0 (0)		1 (6)		1 (3)		2 (13)		4 (22)		6 (18)
Alcohol use disorder severity^d^, n (%)													
	Mild (2-3)	1 (7)		0 (0)		1 (3)		—^e^		—		—	
	Moderate (4-5)	2 (13)		0 (0)		2 (6)		—		—		—	
	Severe (6+)	12 (80)		18 (100)		30 (91)		—		—		—	
Any drug use in the past 60 days^f^, n (%)			13 (87)		12 (67)		25 (76)		—		—		—
Participated in 12-step or self-help group in past 2 months, n (%)			2 (13)		4 (22)		6 (18)		—		—		—
Received outpatient treatment in the past 2 months, n (%)			1 (7)		1 (6)		2 (6)		—		—		—
Received inpatient treatment in the past 2 months, n (%)			1 (7)		1 (6)		2 (6)		—		—		—

^a^A-CHESS: Addiction version of the Comprehensive Health Enhancement Support System.

^b^P-CHESS: Partner version of the Comprehensive Health Enhancement Support System.

^c^Participant checked any 2 or more of the race/ethnicity designations in the survey.

^d^Calculated from the Diagnostic and Statistical Manual of Mental Disorders, Fifth Edition alcohol use disorder checklist, a self-report measure.

^e^Not applicable.

^f^Does not include alcohol, tobacco, or medications used as prescribed by a doctor.

### Intervention Use

**Figure 2:**
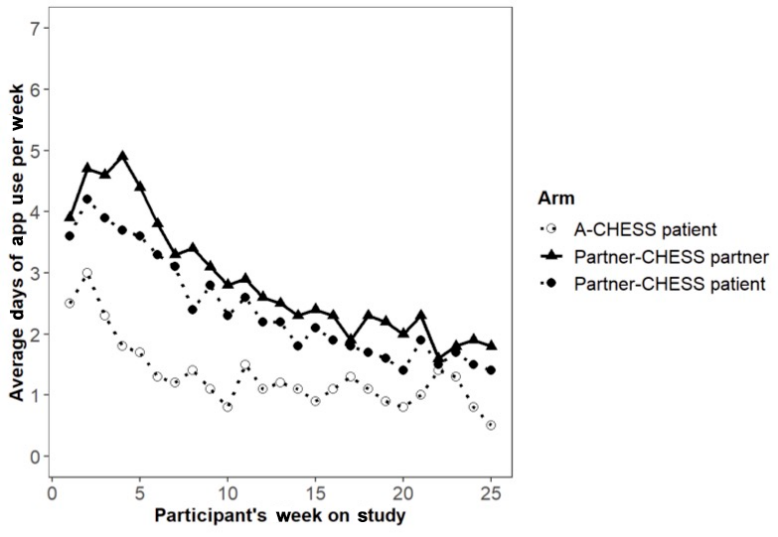
Average days of app use per week across all participants by study arm. A-CHESS: Addiction version of the Comprehensive Health Enhancement Support System; Partner-CHESS: Partner version of the Comprehensive Health Enhancement Support System.

A-CHESS patients used their app a mean of 1.7 (SD 0.7) days per week in the first half (12 weeks) of the study and 1 (SD 0.2) day per week in the second half; Partner-CHESS patients used their app an average of 3.2 (SD 0.6) days per week in the first half and 1.8 (SD 0.3) days per week in the second half; and Partner-CHESS partners used their app an average of 3.8 (SD 0.8) days per week in the first half and 2.1 (SD 0.3) days per week in the second half. As shown in Figure 2, app use days generally declined over time in both study arms. Descriptively, patients in the Partner-CHESS arm used the app on more days than patients in the A-CHESS arm in the first half of the study and, to a lesser extent, in the second half. Within the Partner-CHESS arm, partners generally used the app on more days than patients.

Of the 51 participants who received an app (all 33 patients and the 18 Partner-CHESS partners), 47 (92%) were using their app at the study midpoint and 37 (73%) were using it during the final month.

In exit interviews at 6 months, patients in both study arms and partners in the Partner-CHESS arm rated how helpful they found the app overall and specific features on a 5-point Likert scale (1=not at all, 5=extremely). Means, SD, and n (%) rating 3 or above are reported in Table 2. Overall, the apps were rated as somewhat helpful or better, with patient ratings of A-CHESS (mean 3.1, SD 0.9) being descriptively lower than patient ratings of Partner-CHESS (mean 3.7, SD 1.0) and partner ratings of Partner-CHESS (mean 3.6, SD 0.9) in this sample. For Partner-CHESS features, helpfulness ratings were descriptively highest among partners for the weekly check-in, the e-learning modules, and the discussion group (means >3.5, SDs <1.4) in this sample. Among Partner-CHESS patients, ratings were descriptively highest for the weekly check-in and having their partner participate in the app with them (means 3.7, SDs <1.5). In contrast to Partner-CHESS partners, Partner-CHESS patients gave relatively low ratings to the e-learning modules mean 2.7 (SD 1.5) in this sample.

**Table 2:** A-CHESS^a^ patient, Partner-CHESS^b^ patient, and Partner-CHESS partner ratings of app helpfulness at the end of the study. Helpfulness was rated on a 5-point Likert scale (1=not at all helpful, 5=extremely helpful).

Helpfulness			A-CHESS patients (n=15)				Partner-CHESS patients (n=18)				Partner-CHESS partners (n=18)		
			Rating, mean (SD)		Rated 3 or higher, n (%)	Rating, mean (SD)		Rated 3 or higher, n (%)		Rating, mean (SD)			Rated 3 or higher, n (%)
How helpful was...													
	App overall	3.1 (0.9)		13 (87)		3.7 (1.0)		16 (89)	3.6 (0.9)			16 (89)	
	Daily check-in	—^c^		—		3.2 (1.3)		13 (72)	3.1 (1.2)			11 (61)	
	Weekly check-in	3.4 (1.4)		12 (80)		3.7 (1.2)		15 (83)	3.7 (1.0)			15 (83)	
	e-learning modules	—		—		2.7 (1.5)		10 (56)	3.6 (1.2)			14 (78)	
	PartnerLink (sending prepopulated or custom text messages to your partner from the app)	—		—		1.8 (1.2)		4 (22)	1.4 (0.7)			2 (11)	
	Having your partner participate in using the app with you	—		—		3.7 (1.4)		14 (78)	3.1 (1.4)			12 (67)	
	Addressing other concerns like taking care of yourself and your relationship	2.9 (1.2)		9 (60.0)		3.4 (1.3)		14 (78)	3.4 (1.2)			14 (78)	
	Using the discussion group on the app	3.1 (1.1)		11 (73.3)		3.1 (1.1)		13 (72)	3.6 (1.3)			13 (72)	
	Using relationship-related parts of the app (cravings discussion, trigger list, etc)	—		—		2.8 (1.4)		10 (56)	2.8 (1.2)			12 (67)	

^a^A-CHESS: Addiction version of the Comprehensive Health Enhancement Support System.

^b^Partner-CHESS: Partner version of the Comprehensive Health Enhancement Support System.

^c^Not available

### Primary Outcomes

**Figure 3:**
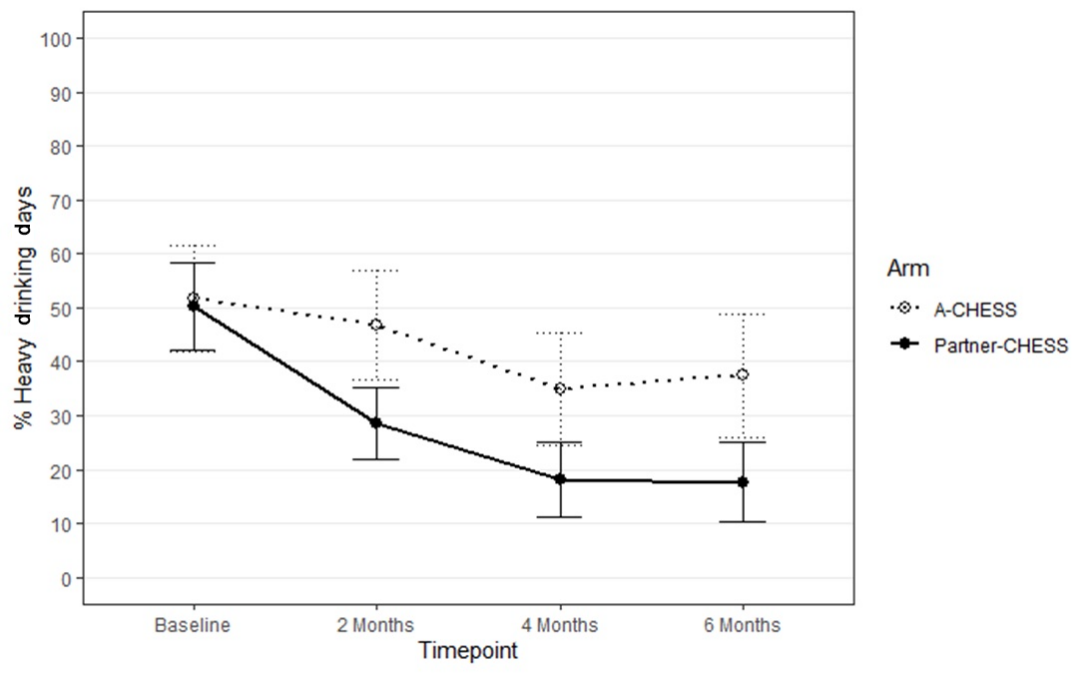
Percent heavy drinking days by study arm over time. Points represent the observed mean for percent drinking days; lines connecting points are added only to aid visualization of the observed mean pattern. Error bars are the simple SE for each mean. A-CHESS: Addiction version of the Comprehensive Health Enhancement Support System; Partner-CHESS: Partner version of the Comprehensive Health Enhancement Support System.

The main effect of the study arm on percent heavy drinking days was not significant (β=–17.4, 95% CI –36.1 to 1.4; *P*=.07), but as shown in Figure 3, there was a descriptively lower percentage of heavy drinking days in the Partner-CHESS versus A-CHESS arm across intervention time points (for descriptive statistics, see Table S1 in the Multimedia Appendix 1). The study arm time point interaction was not significant (β=–0.8, 95% CI –11.8 to 10.2; *P*=.88), indicating that the magnitude of the study arm effect did not meaningfully change over time. Effect sizes for the simple effects of the study arm on percent heavy drinking days were all moderate across the 3 postintervention time points (2 months: Hedges *g*=–0.66, 95% CI –1.34 to 0.03; 4 months: Hedges *g*=–0.54, 95% CI –1.22 to 0.15; 6 months: Hedges *g*=–0.53, 95% CI –1.22 to 0.16).

**Figure 4:**
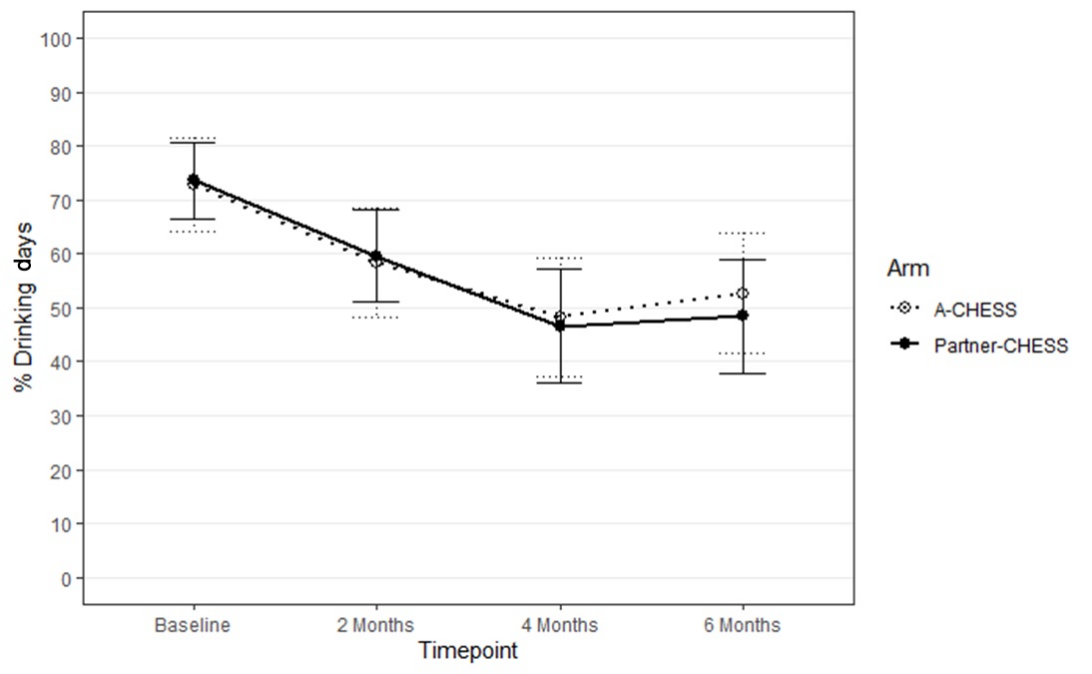
Percent drinking days by study arm over time. Points represent the observed mean for percent drinking days; lines connecting points are added only to aid visualization of the observed mean pattern. Error bars are the simple SE for each mean. A-CHESS: Addiction version of the Comprehensive Health Enhancement Support System; Partner-CHESS: Partner version of the Comprehensive Health Enhancement Support System.

As shown in Figure 4, there was no significant main effect of the study arm for our other primary outcome, percent drinking days (β=–2.1, 95% CI –24.8 to 20.7; *P*=.85), and percent drinking days was descriptively similar for both interventions across intervention time points (see Table S1 in Multimedia Appendix 1 for descriptive statistics). Furthermore, the magnitude of the study arm effect on percent drinking days did not change over time (study arm time point; β=–2.8, 95% Cl –15.4-9.9; *P*=.65). Effect sizes for the simple effects of study arm on percent drinking days were less than small across time points (2 months: Hedges *g*=0.02, 95% CI –0.64 to 0.69; 4 months: Hedges *g*=–0.06, 95% CI –0.72 to 0.61; 6 months: Hedges *g*=–0.53, 95% CI –0.78 to 0.54).

We limited our analyses of the primary outcomes to the effects of the study arm (ie, study arm and study arm time point effects) to increase statistical validity. However, we recognize the main effect of the time point on the 2 outcomes may be of interest to some readers and therefore report the time point main effects here for completeness. Specifically, the main effect for time point was not significant for either percent heavy drinking days (β=–5.0, 95% Cl –10.5-0.5; *P*=.07) or percent drinking days (β=–4.2, 95% Cl –10.5-2.1; *P*=.18)

### Secondary Outcomes

Baseline-adjusted means and SDs by study arm are reported for all secondary outcomes, separately for patients and partners, in Multimedia Appendix 1. Table S2 in Multimedia Appendix 1 also includes effect sizes for simple effects of the study arm at each time point. We did not conduct formal statistical analyses on these secondary outcomes due to the large number of potential tests. However, we observed beneficial effects of at least moderate size for partners in the Partner-CHESS arm (relative to A-CHESS) for peer support, emotional coping support, and instrumental coping support. Conversely, patients in the Partner-CHESS arm displayed detrimental effects of at least moderate size for psychological distress and relationship satisfaction.

## Discussion

### Findings and Implications

This pilot study enabled us to establish the feasibility and protocols for a subsequent larger RCT. Further, the preliminary analyses of study arm effects on primary outcomes motivate similar analyses with a larger sample size of participants and greater statistical power. Building on A-CHESS, a patient-only app already shown to improve outcomes among individuals with AUD, our ultimate aim is to assess the potential for a couple-focused version of the app to improve patient outcomes relative to the patient-only version.

A critical finding was that the novel and previously untested intervention, Partner-CHESS, was used by participants throughout the study and rated helpful. In both arms, app use decreased over time, but this is typical of apps in general and mobile health (mHealth) in particular. For health apps in the real world, attrition has been pegged at more than 97% by day 30 [65]. A more direct comparison is a review of controlled and observational studies of app-based interventions targeting a range of chronic diseases, which found an average dropout rate of 43% [66]. Retention was notably better than this for both app conditions in this study.

Among patients in the sample, Partner-CHESS showed more use per week than A-CHESS, especially in the first half, and Partner-CHESS patients gave higher ratings of overall helpfulness. Meanwhile, Partner-CHESS partners in the sample used their app more than any other group in the study. Their usage may indicate a general pattern whereby partners are highly motivated to engage in an intervention to reduce their partner’s drinking, or it may reflect the fact that 72% (13/18) of Partner-CHESS partners were female. Women tend to assume caregiver roles in relationships [67], and there is some evidence that they show higher rates of adherence to online psychological interventions [68], although a 2022 study found that men (rather than women) were more likely to use online support for recovery from AUD [69]. Given the small sample size, we cannot disentangle gender and treatment arm, but it is noteworthy that Partner-CHESS patients (mostly men) and partners (mostly women) showed equivalent ratings of how helpful the intervention was.

Our ongoing, large RCT will allow us to formally test for study arm differences controlling for gender and, if confirmed, explore reasons for more app use among Partner-CHESS versus A-CHESS patients. For example, a within-couple correlation may reveal that Partner-CHESS patients were influenced to increase their app use when their partners used it more. Engagement rates described in this pilot study should be interpreted with caution and as descriptive statistics related to the study sample (ie, not as results that may reflect general differences in engagement across larger patient populations) due to the small sample size that precluded significance testing.

Of the 33 couples in the study, all but 2 were recruited through social media and email rather than treatment clinics. Our final sample therefore comprised nontreatment-seeking individuals who met criteria for AUD rather than patients in treatment, along with their romantic partners. Nonetheless, we found that online methods were a feasible approach to enrolling romantic dyads in a clinical trial testing mHealth for AUD, as recruitment proceeded with no delay. Further, retention was high: only 1 dyad was lost after assignment to condition but before the period of app use, and none were lost after app use began.

This experience has implications for both clinical research and public health. First, for research purposes, recruitment through methods such as social media and email offers the possibility of reaching vastly wider geographic areas and greater numbers of dyads, fine-tuning recruitment efforts to target subgroups (eg, gender, race, ethnicity, and age), and allocating study resources such as recruitment expenses and staff time with greater flexibility. Second, these methods enabled us to engage individuals who were not in treatment. Currently, more than 90% of US adults with diagnosable AUD do not seek treatment for it in their lifetime [6], a dismal statistic. Online and media outreach may address issues of access to treatment, including stigma and even general awareness of AUD, and help ameliorate this public health crisis.

In addition to examining the feasibility of this pilot study, we conducted preliminary analyses of primary drinking outcomes to inform future research. Although the main effect of the study arm on percent heavy drinking days was not significant (*P*=.07), the preliminary analyses motivate further comparative effectiveness testing with a larger sample size of participants and greater statistical power. In contrast, there was no evidence that Partner-CHESS reduced the percentage of drinking days at any point. This suggests that for individuals with AUD who have willing and able romantic partners, involving those partners in the recovery process with Partner-CHESS may have the potential to help reduce the quantity of alcohol consumed on drinking days, that is, heavy drinking, but not necessarily the frequency of drinking days. However, not all individuals with AUD have a romantic partner or 1 who will engage with mHealth tools to support their recovery. For these patients, A-CHESS remains an effective tool to reduce the risk of relapse after treatment [21,70]. In addition, future work may look to configuring Partner-CHESS for a wider array of dyads involving other family members or close friends. We caution that all results from this pilot should be considered preliminary because of the small sample size.

### Limitations

As a pilot, this study is underpowered, so the results of all analyses must be considered preliminary and in need of replication. Participants received payment for surveys and were provided with smartphones and data plans during the study; although these incentives were small and of short duration, they may have introduced biased in-app use results. Finally, patients were primarily male 76% (25/33), partners were primarily female 79% (26/33), and 80% (53/66) of participants were White. Recruitment efforts tailored for greater diversity in race and ethnicity and dyads’ gender configurations (eg, female patients with male partners and same-gender couples) would increase the generalizability of results. In light of successful recruitment with online and media advertising, and the availability of analytics to target specific populations through media communications, we expect to increase diversity in future studies.

### Conclusions

This pilot study provides support and suggests promising directions for further study involving partners of individuals in recovery from AUD. Although it was not powered to determine conclusive effects of Partner-CHESS versus A-CHESS, preliminary results motivate additional, well-powered studies to determine if Partner-CHESS may help reduce heavy drinking. In addition, the successful recruitment of participants with AUD from the general population through online methods has positive implications for both research efforts and public health. Combined with substantial previous evidence of clinical benefits derived from A-CHESS [21,23,24,70], these preliminary findings suggest the potential for technology such as Partner-CHESS and A-CHESS to help those who are not in traditional treatment for AUD, an important implication given the continued lack of access to treatment, including reluctance to seek it. A fully powered RCT to test drinking and relationship outcomes among dyads is currently underway.

## Appendix

Multimedia Appendix 1 Baseline-adjusted mean scores and effect sizes for primary and secondary outcome measures by study arm and time point, including psychosocial outcomes, relationship satisfaction, family environment, psychological distress, sobriety motivation, peer support, and coping strategies for patients and partners.

Multimedia Appendix 2 CONSORT-EHEALTH (v 1.6.1) checklist.

## Abbreviations

ABCT: Alcohol Behavioral Couple Therapy
A-CHESS: Addiction version of the Comprehensive Health Enhancement Support System
AUD: alcohol use disorder
CONSORT: Consolidated Standards of Reporting Trials
DSM-5: Diagnostic and Statistical Manual of Mental Disorders, Fifth Edition
IRB: Institutional Review Board
mHealth: mobile health
NIAAA: National Institute on Alcohol Abuse and Alcoholism
Partner-CHESS: Partner version of the Comprehensive Health Enhancement Support System
RCT: randomized controlled trial
