# Supplementary material for: Couple-Focused Smartphone Intervention to Reduce Problem Drinking: Pilot Randomized Control Trial
Source: JMIR Form Res. 2024 Nov 1;8:e58622. doi: 10.2196/58622 (PMC11568395; doi:10.2196/58622)
Supplement: Multimedia Appendix 1 [file formative_v8i1e58622_app1.docx]

# Multimedia Appendix

## Primary Outcomes: Descriptive Statistics

Table S1. Baseline-adjusted mean scores on primary outcome measures by study arm over time.

| Outcome by timepoint | A-CHESS  (n=15 dyads)  M (SD) | Partner-CHESS  (n=18 dyads)  M (SD) | Hedges *g* | 95% confidence interval |
| --- | --- | --- | --- | --- |
| % heavy drinking days |  |  |  |  |
| 2 months | 46.2 (24.1) | 28.9 (26.9) | –0.66 | –1.34 to 0.03 |
| 4 months | 34.5 (30.5) | 18.6 (27.3) | –0.54 | –1.22 to 0.15 |
| 6 months | 37.0 (37.8) | 18.0 (31.2) | –0.53 | –1.22 to 0.16 |
| % drinking days |  |  |  |  |
| 2 months | 58.6 (26.5) | 59.4 (32.8) | 0.02 | –0.64 to 0.69 |
| 4 months | 48.5 (34.2 | 46.4 (39.4) | –0.06 | –0.72 to 0.61 |
| 6 months | 53.0 (33.6) | 48.2 (42.9) | –0.12 | –0.78 to 0.54 |

## Secondary Outcomes

### Measures

At each timepoint (baseline, 2, 4, and 6 months), psychosocial outcomes related to relatedness, motivation, and coping were assessed. Measures were selected for good psychometric properties with similar populations and because they represent pertinent intervention targets and hypothesized mechanisms of change.

#### Relationship Satisfaction

Patients' and partners’ relationship satisfaction was assessed with the 7-item Dyadic Adjustment Scale–Brief (DAS-7), rating agreement between partners on 3 values-based items such as "philosophy of life" (0=always disagree, 5=always agree), frequency of 3 positive interactions such as "calmly discuss something together" (0=never, 5=more than once a day), and a single item "degree of happiness, all things considered, of your relationship" (0=extremely unhappy, 6=perfect).[46–48] Responses were summed; the total possible range was 0–36, with higher scores indicating better couple functioning, and scores of 21 or more indicating a "nondistressed" relationship. Cronbach’s α=.84–.90 across the 4 timepoints.

#### Perceptions of Family Environment

Patients and partners both completed the 9-item Cohesion subscale of the Family Environment Scale, with true–false statements regarding, for example, feelings of togetherness and helping around the home, as well as the 9-item Conflict subscale, with true–false statements regarding anger and fighting.[49] The two subscales were summed separately, following scoring methods in the scale manual. The total possible range for Cohesion was 4–65 and the range for Conflict was 33–80, with higher scores indicating, respectively, greater cohesion or conflict. Cronbach’s α=.80–.90 across the 4 timepoints.

#### Psychological Distress

Patients' and partners’ psychological distress was assessed with the 45-item Outcome Questionnaire (OQ-45), rating frequency over the past week for positive statements such as "I am a happy person" (0=almost always, 4=never) and negative statements such as "I feel irritated" (0=never, 4=almost always).[50] Responses were summed; the total possible range was 0–180, with higher scores indicating more distress (anxiety, depression, somatic problems, stress), greater difficulty with interpersonal relationships and social roles, and lower quality of life. Scores of 63 or more indicate symptoms of clinical significance, and changes of 14 points or more are considered reliable.[51] Cronbach’s α=.95–.96 across the 4 timepoints.

#### Sobriety Motivation

Patients completed the 5-item Commitment to Sobriety Scale, rating agreement with such statements as "Staying sober is the most important thing in my life" and "I am totally committed to staying off alcohol/drugs" (1=strongly disagree, 5=strongly agree).[52] Responses were summed; the total possible range was 5–25, with higher scores suggesting greater commitment. Cronbach’s α=.88–.95 across the 4 timepoints.

#### Partner’s Peer Support

Partners reported on the availability of peer support using the 5-item McTavish Bonding Scale, indicating how often statements were true (eg, "I am building a bond with others who have a partner dealing with substance abuse," 1=never, 5=almost always).[53] Items were summed; the total possible range was 5–25, with higher scores suggesting more support. Cronbach’s α=.83–.90 across the 4 timepoints.

#### Partner’s Coping Strategies

Partners responded to 18 items from the Partner Interaction Questionnaire, adapted for drinking rather than smoking.[54,55] Two items not transferrable to drinking were dropped. Items rated the frequency (0=never, 4=very often) of behavioral strategies over the past 30 days in four categories [56]: 7 emotional support items (eg, "Complimented them on not drinking," total possible range=0–28); 4 instrumental support items (eg, "Helped them think of substitutes for drinking," total possible range=0–16); 6 complaints about drinking (eg, "Asked them to quit drinking," total possible range=0–24); and 2 items critical of patient (eg, "Commented on their lack of willpower," total possible range=0–8). Higher scores in the 4 categories indicate, respectively, more emotional support, instrumental support, complaints, and criticism. Emotional and instrumental support are considered positive coping strategies consistent with modules in Partner-CHESS, while complaints and criticism are considered poor coping strategies. The 4 categories were assessed separately. Cronbach’s α=.88–.91 across the 4 timepoints.

### Descriptive Analyses of Secondary Outcomes

Table S2 shows baseline-adjusted means and standard deviations by study arm, separately for patients and partners, as well as effect sizes for simple effects of study arm at each timepoint.

Given the dyadic focus of the Partner-CHESS intervention, 3 secondary outcomes were assessed to explore the possible impact of study arm on the couple’s relationship (relationship satisfaction, family environment cohesion, family environment conflict). As shown in Table S1, simple effects of study arm for all 3 outcomes for both patient and partner were consistently nonsignificant and were generally small. The few medium size effects (eg, partners’ ratings of relationship satisfaction at 6 months, conflict at 4 months) showed no clear pattern suggesting positive or negative effects of Partner-CHESS versus A-CHESS.

Additional secondary outcomes for the patient were psychological distress and sobriety motivation. Effects of study arm at each timepoint were consistently nonsignificant and generally small, with no descriptively clear pattern of changes in one study arm versus the other.

Additional secondary outcomes for the partner included psychological distress, peer support, and 2 positive coping scales (emotional and instrumental support) and 2 negative coping scales (complaints and critical about patient). The results suggest some benefits of Partner-CHESS (vs. A-CHESS) for partners’ perceptions of support. There were large, significant effect sizes favoring Partner-CHESS at 2, 4, and 6 months for peer support; large, significant effect sizes at 2 and 4 months for instrumental support; and a medium, nonsignificant effect size at 2 months for emotional support. Study arm comparisons for psychological distress and the 2 negative coping styles showed small, nonsignificant effect sizes at all timepoints.

Table S2. Baseline-adjusted mean scores on secondary outcome measures by study arm over time.

| Outcome (possible score range) by timepoint | A-CHESS (n=15 dyads)  M (SD) | Partner-CHESS (n=18 dyads)  M (SD) | Hedges *g* | 95% confidence interval |
| --- | --- | --- | --- | --- |
| Patients | | | | |
| Relationship satisfaction (0–36) |  |  |  |  |
| 2 months | 23.4 (2.7) | 23.6 (4.7) | 0.05 | –0.60 to 0.70 |
| 4 months | 25.4 (4.0) | 24.6 (5.3) | –0.16 | –0.82 to 0.51 |
| 6 months | 25.0 (5.3) | 24.0 (4.6) | –0.18 | –0.85 to 0.49 |
| Family environment: cohesion (4–65) |  |  |  |  |
| 2 months | 51.7 (6.3) | 49.9 (9.5) | –0.22 | –0.87 to 0.44 |
| 4 months | 57.3 (5.4) | 54.4 (11.1) | –0.32 | –0.97 to 0.33 |
| 6 months | 55.9 (8.3) | 54.3 (12.5) | –0.15 | –0.80 to 0.51 |
| Family environment: conflict (33–80) |  |  |  |  |
| 2 months | 41.9 (10.6) | 45.1 (9.1) | 0.32 | –0.36 to 0.99 |
| 4 months | 41.9 (6.4) | 43.2 (9.6) | 0.16 | –0.50 to 0.81 |
| 6 months | 43.8 (11.0) | 43.4 (8.1) | –0.03 | –0.70 to 0.64 |
| Psychological distress (0–180) |  |  |  |  |
| 2 months | 50.4 (15.0) | 57.2 (21.8) | 0.35 | –0.32 to 1.01 |
| 4 months | 39.5 (27.5) | 53.7 (19.1) | 0.58 | –0.12 to 1.27 |
| 6 months | 47.9 (26.5) | 45.5 (17.8) | –0.10 | –0.77 to 0.58 |
| Sobriety motivation  (5–25) |  |  |  |  |
| 2 months | 14.4 (3.6) | 14.2 (4.7) | –0.03 | –0.69 to 0.63 |
| 4 months | 14.8 (4.2) | 16.8 (4.5) | 0.44 | –0.23 to 1.12 |
| 6 months | 14.9 (4.6) | 16.4 (4.2) | 0.33 | –0.35 to 1.00 |
| Partners | | | | |
| Relationship satisfaction (0–36) |  |  |  |  |
| 2 months | 23.8 (4.0) | 22.9 (4.8) | –0.19 | –0.86 to 0.47 |
| 4 months | 23.6 (3.5) | 21.8 (5.7) | –0.37 | –1.02 to 0.30 |
| 6 months | 24.0 (3.5) | 21.0 (7.4) | –0.52 | –1.18 to 0.15 |
| Family environment: cohesion (4–65) |  |  |  |  |
| 2 months | 51.4 (11.3) | 53.2 (12.5) | 0.14 | –0.52 to 0.81 |
| 4 months | 55.6 (10.4) | 57.4 (9.2) | 0.18 | –0.50 to 0.85 |
| 6 months | 56.4 (11.7) | 53.1 (15.9) | –0.22 | –0.88 to 0.44 |
| Family environment: conflict (33–80) |  |  |  |  |
| 2 months | 47.0 (9.3) | 43.3 (8.8) | –0.39 | –1.07 to 0.29 |
| 4 months | 45.2 (8.2) | 41.1 (8.2) | –0.48 | –1.16 to 0.20 |
| 6 months | 44.6 (7.5) | 44.4 (9.1) | –0.03 | –0.69 to 0.64 |
| Psychological distress (0–180) |  |  |  |  |
| 2 months | 57.1 (17.5) | 54.0 (13.0) | –0.19 | –0.86 to 0.49 |
| 4 months | 48.7 (11.2) | 53.7 (22.9) | 0.27 | –0.39 to 0.91 |
| 6 months | 51.1 (18.4) | 55.6 (22.9) | 0.21 | –0.45 to 0.87 |
| Peer support (5–25) |  |  |  |  |
| 2 months | 12.9 (5.7) | 17.3 (2.9) | 0.92 | 0.18 to 1.64 |
| 4 months | 11.4 (3.8) | 15.8 (3.7) | 1.14 | 0.40 to 1.86 |
| 6 months | 12.8 (3.5) | 16.6 (4.8) | 0.88 | 0.18 to 1.57 |
| Coping: emotional support (0–28) |  |  |  |  |
| 2 months | 12.9 (4.1) | 15.3 (5.0) | 0.51 | –0.17 to 1.18 |
| 4 months | 12.5 (4.9) | 13.8 (3.9) | 0.29 | –0.39 to 0.96 |
| 6 months | 12.3 (3.8) | 13.6 (4.9) | 0.29 | –0.38 to 0.95 |
| Coping: instrumental support (0–16) |  |  |  |  |
| 2 months | 5.6 (2.2) | 7.8 (2.6) | 0.90 | 0.19 to 1.59 |
| 4 months | 5.3 (2.7) | 7.7 (3.1) | 0.80 | 0.10 to 1.48 |
| 6 months | 6.2 (2.5) | 7.1 (3.5) | 0.27 | –0.40 to 0.93 |
| Coping: complaints about patient (0–24) |  |  |  |  |
| 2 months | 6.4 (5.6) | 7.0 (3.4) | 0.12 | –0.55 to 0.80 |
| 4 months | 4.0 (3.7) | 5.0 (2.7) | 0.27 | –0.41 to 0.95 |
| 6 months | 5.1 (3.0) | 5.1 (4.5) | –0.02 | –0.67 to 0.64 |
| Coping: critical of patient (0–8) |  |  |  |  |
| 2 months | 1.7 (1.8) | 1.4 (2.0) | –0.14 | –0.80 to 0.53 |
| 4 months | 1.8 (1.7) | 1.5 (2.2) | –0.16 | –0.82 to 0.50 |
| 6 months | 1.6 (1.4) | 1.3 (1.5) | –0.25 | –0.92 to 0.42 |

*Note.* N=33 patient–partner dyads (n=15 A-CHESS patients, 15 A-CHESS partners, 18 Partner-CHESS patients, 18 Partner-CHESS partners). Higher scores=higher values of the outcome variable as stated (eg, more commitment, more conflict).

## References

46. Early Intervention Foundation. Dyadic Adjustment Scale (DAS-7): 7-item report measure. March 2020. Accessed March 6, 2024. https://www.eif.org.uk/files/resources/measure-report-ipr-das-7.pdf

47. Hunsley J, Best M, Lefebvre M, Vito D. The seven-item short form of the Dyadic Adjustment Scale: Further evidence for construct validity. Am J Fam Ther. 2001 Oct 1;29(4):325-335. doi:10.1080/01926180126501.

48. Sharpley CF, Rogers HJ. Preliminary validation of the Abbreviated Spanier Dyadic Adjustment Scale: Some psychometric data regarding a screening test of marital adjustment. Educ Psychol Meas. 1984;44(4):1045–1049. doi:10.1177/00131644844440.

49. Moos RH, Moos BS. Family Environment Scale Manual: Development, Applications and Research. 4th ed. Mind Garden; 2009.

50. Lambert MJ, Burlingame GM, Umphress V, et al. The reliability and validity of the Outcome Questionnaire. Clin Psychol Psychother. 1996;3(4):249-258. doi:10.1002/(SICI)1099-0879(199612)3:4<249::AID-CPP106>3.0.CO;2-S.

51. Lambert MJ. Scoring and interpreting the Outcome Questionnaire-45. August 17, 1998. Accessed March 12, 2024. http://www.ehrs.com/forms/pei/oq45scoreguide.pdf

52. Kelly JF, Greene MC. Beyond motivation: Initial validation of the Commitment to Sobriety scale. J Subst Abuse Treat. 2014;46(2):257-263. doi:10.1016/j.jsat.2013.06.010. PMID: **23953168.**

53. Namkoong K, DuBenske LL, Shaw BR, et al. Creating a bond between caregivers online: Effect on caregivers' coping strategies. J Health Commun. 2012;17(2):125-140. doi:10.1080/10810730.2011.585687. PMID: **22004055.**

54. Cohen S, Lichtenstein E. Partner behaviors that support quitting smoking. J Consult Clin Psychol. 1990;58(3):304-309. doi:10.1037//0022-006x.58.3.304. PMID: 2365893.

55. Osman A, Amodei N, Lamb RJ. Further psychometric analysis of the 20-item Partner Interaction Questionnaire in an adult sample of smokers. Psychol Addict Behav. 2019;33(6):567-573. doi:10.1037/adb0000477. PMID: **31192622.**

56. Burns RJ, Rothman AJ, Fu SS, Lindgren B, Joseph AM. The relation between social support and smoking cessation: Revisiting an established measure to improve prediction. Ann Behav Med. 2014;47(3):369-375. doi:10.1007/s12160-013-9558-7. PMID: **24222508.**
